# Drug-drug interactions between sex hormones and analgesics linked to thromboembolic events: an FDA adverse event reporting system analysis

**DOI:** 10.3389/fphar.2026.1791198

**Published:** 2026-04-21

**Authors:** Seiki Yamazaki, Kenji Onda, Koichi Masuyama

**Affiliations:** 1 Regulatory Science laboratory, School of Pharmacy, Tokyo University of Pharmacy and Life Sciences, Tokyo, Japan; 2 Department of Clinical Pharmacology, School of Pharmacy, Tokyo University of Pharmacy and Life Sciences, Tokyo, Japan

**Keywords:** analgesics, arterial thromboembolism (ATE), drug–drug interactions (DDI), FDA adverse event reporting system (FAERS), female hormone, male hormone, non-steroidal anti-inflammatory drugs (NSAIDs), venous thromboembolism (VTE)

## Abstract

**Introduction:**

Thromboembolic events, including venous thromboembolism (VTE) and arterial thromboembolism (ATE), are affected by numerous pharmacological factors. Although sex hormones and analgesics have been individually associated with thrombotic risk, the clinical relevance of potential drug–drug interactions (DDIs) between these agents remains unclear. This study examines how DDIs affect reports of thrombosis, including ATE and VTE, and compares them with existing epidemiological data.

**Methods:**

A total of 15.9 million reports from the Food and Drug Administration Adverse Event Reporting System (FAERS [JAPIC FAERS]) were evaluated to assess potential DDIs between sex hormones (female and male) and analgesics in relation to VTE and ATE. Disproportionality analyses were conducted using crude reporting odds ratios (cRORs) and DDI signal detection via four complementary statistical methods.

**Results:**

Among the cases reviewed, 162,846 patients experienced VTE, and 283,197 experienced ATE. Both sex hormones and analgesics demonstrated significant associations with increased reporting of VTE and ATE. Notably, in the DDI analysis between female hormones and analgesics, thirteen drug pairs were positive across all four algorithms for VTE, whereas only one pair met this criterion for ATE. No consistent DDI signals were identified for male hormones in either VTE or ATE cases.

**Discussion:**

Although spontaneous reporting systems have inherent limitations, this study’s findings suggest sex-specific differences in the impact of concomitant analgesic use on thromboembolic events. The observed increase in VTE reporting with the combined use of female hormones and analgesics aligns with existing epidemiologic data and underscores the utility of FAERS-based approaches for identifying clinically relevant DDIs.

## 1 Introduction

Recently, the overall use of prescription medications and prevalence of polypharmacy have markedly increased (Masnoon et al., 2017; Wang et al., 2023). Consequently, the concomitant use of multiple therapeutic agents has become increasingly common, raising concerns about altered plasma drug concentrations and pharmacodynamic interactions that may lead to unexpected adverse events due to drug–drug interactions (DDIs).

While pre-marketing randomized controlled trials serve as the primary avenue for assessing drug safety, these studies are typically limited by small sample sizes and often fail to comprehensively evaluate DDIs. Accordingly, DDIs have become a significant concern within postmarketing clinical settings.

Spontaneous reporting systems (SRSs) represent a key source of post-marketing pharmacovigilance data. Adverse event reports collected through SRSs capture real-world drug utilization and are instrumental for the early detection of DDI-related adverse events, including those related to previously undocumented interactions (Banda et al., 2016). In response to this need, several statistical methodologies for detecting DDI signals in SRS data have been developed, along with analytical models (Noguchi et al., 2019).

The Food and Drug Administration (FDA) Adverse Event Reporting System (FAERS) is among the largest SRS databases worldwide. FAERS offers a comprehensive dataset grounded in clinical practice and is particularly advantageous for investigating rare adverse events (Sakaeda et al., 2013). With its extensive coverage of drugs and adverse events, FAERS has been widely used in studies analyzing rare adverse events during the post-marketing phase.

Various methods have been suggested for detecting signals of drug-induced adverse events, and are commonly applied in pharmacovigilance practice (Bate and Evans, 2009). Furthermore, FAERS data have been used to analyze potential DDIs based on reports of concomitant drug use, as well as to identify drugs that may mitigate drug-induced adverse events (Zhao et al., 2013).

However, SRSs have inherent limitations, including the inability to estimate true incidence rates, susceptibility to reporting biases, and multiple confounding factors. Hence, directly comparing reporting odds ratios (RORs) from disproportionality analyses is considered inappropriate. To address these challenges, adjusted reporting odds ratios (aRORs), calculated via logistic regression under specific conditions, have been proposed for DDI analysis using SRS (Oshima et al., 2018; Zamami et al., 2019; Onda et al., 2023). Additionally, several models have been developed to detect DDI signals between two drugs, including the Ω shrinkage measure model (Norén et al., 2006; Norén et al., 2008), additive and multiplicative models (Thakrar et al., 2007), and the combination risk ratio (CRR) model (Susuta and Takahashi, 2014). Among these, the Ω shrinkage measure model—adopted by the World Health Organization (WHO) Uppsala Monitoring Centre—evaluates disproportionality by comparing observed to expected values and is considered the most conservative approach (Noguchi et al., 2020).

Thrombosis encompasses venous thromboembolism (VTE), deep vein thrombosis (DVT), pulmonary embolism (PE), and arterial thromboembolism (ATE), which can manifest as myocardial infarction or stroke. As a significant public health concern, thrombosis accounts for approximately one-quarter of global mortality (GBD 2019 Diseases and Injuries Collaborators, 2020). Over the past 2 decades, age-adjusted mortality rates associated with ATE have declined in high-income countries, whereas VTE-related mortality rates have remained stable. However, this trend shifted markedly following the onset of the COVID-19 pandemic, with both ATE- and VTE-related mortality increasing in the past 5 years (Sidney et al., 2022).

Thrombosis is recognized as a potential adverse event of pharmacotherapy. Although labeling requirements differ internationally, package inserts frequently identify numerous drugs associated with thrombosis risk, including hormone therapies, antirheumatic agents, antipsychotics, anticancer drugs, lipid-lowering agents, anti-HIV drugs, nonsteroidal anti-inflammatory drugs (NSAIDs), and corticosteroids. Nonetheless, the current evidence base regarding the thrombosis risk associated with combined drug use remains insufficient.

Female hormonal therapies and NSAIDs are both routinely used, for example, in the management of dysmenorrhea and other common menstrual disorders, making their concomitant use and potential DDIs clinically relevant. Dysmenorrhea is highly prevalent among adolescent and young adult women, and standard management often involves repeated or prolonged use of NSAIDs, hormonal therapies, or their combination (Author anonymus, 2018; McKenna and Fogleman, 2021). Despite the widespread use of these agents, evidence regarding the thromboembolic safety of their concomitant use remains limited. Evaluation of rare but serious adverse outcomes, such as VTE, particularly in the context of potential DDIs, requires large-scale data sources capable of detecting infrequent events. SRSs including FAERS, are therefore well suited for pharmacovigilance analyses aimed at identifying potential DDI-related safety signals.

The objective of the current study was to assess the impact of DDIs on reported cases of thrombosis, including ATE and VTE, and to compare these findings with existing epidemiological literature. Specifically, we examined the trend of increased thrombosis reports associated with the combined use of sex hormones (female and male) and analgesics (NSAIDs and acetaminophen). We used FAERS database to analyze reports of VTE and ATE, calculating crude RORs (cRORs) for drugs associated with these adverse events. Subsequently, we examined DDIs in cases involving the concomitant use of sex hormones and analgesics, using a 4 × 2 contingency table and four statistical measures to evaluate the effects of these combinations.

## 2 Materials and methods

### 2.1 Data source and mining

The analysis utilized JAPIC FAERS (FAERS pre-processed by the Japan Pharmaceutical Information Center), which is based on FAERS data spanning from the fourth quarter of 1997 to the first quarter of 2024. The dataset was curated by eliminating duplicate entries and standardizing drug names. In the JAPIC FAERS dataset, duplicate records were identified primarily using PRIMARYID and CASEID. Records sharing either identifier were screened for potential duplication. When records with identical PRIMARYID or CASEID differed in key variables (age, sex, or adverse event onset date), duplication was assessed based on additional information, including body weight and manufacturer report number. Conversely, records without matching PRIMARYID or CASEID were also evaluated for potential duplication using manufacturer report number in combination with relevant clinical variables. Records determined to represent the same case were removed from the analysis. As FAERS is an anonymized public database, Institutional Review Board approval was not required. This study followed the READUS-PV (REporting of A Disproportionality analysis for drug Safety signal detection using individual case safety reports in PharmacoVigilance) guidelines (Fusaroli et al., 2024). The study used several FAERS data tables: DEMO (containing age and sex data), DRUG (drug details), REAC (adverse event reports), and INDI (indications). These tables were linked using the primary ID, and all analyses were performed using Microsoft Office 365 Access (Microsoft, Tokyo).

### 2.2 Definition of adverse events

Adverse events were defined using Preferred Terms (PTs) and Standardized MedDRA Queries (SMQs) from MedDRA version 27.0. VTE and ATE corresponded to SMQ codes 20000084 and 20000082, respectively. The PTs used are listed in the Supplementary Material (Supplementary Tables S1, S2). The complete list of drugs included in the disproportionality analysis is provided in the Supplementary Material (Supplementary Table S3).

### 2.3 Disproportionality analysis and drug-drug interactions analysis

**FIGURE 1:**
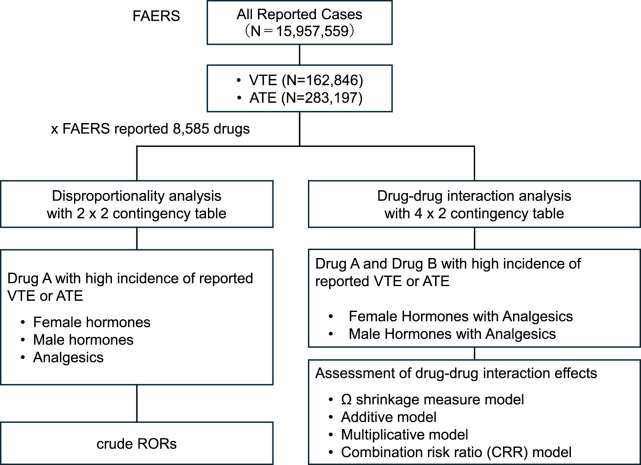
Flow chart summarizing disproportionality analyses of thromboembolic events (VTE and ATE) using FAERS data. Analyses include calculation of cRORs, 95% CIs, and χ^2^ values. Significant signals (lower bound of 95% Cl > 1.0) were identified for female and male hormones, and analgesics. Drug–drug interactions were assessed using four models.

First, a disproportionality analysis was performed by evaluating all reported drug exposures (8,585 distinct agents), with specific extraction of reports related to VTE and ATE from the FAERS database (Figure 1).

**FIGURE 2:**
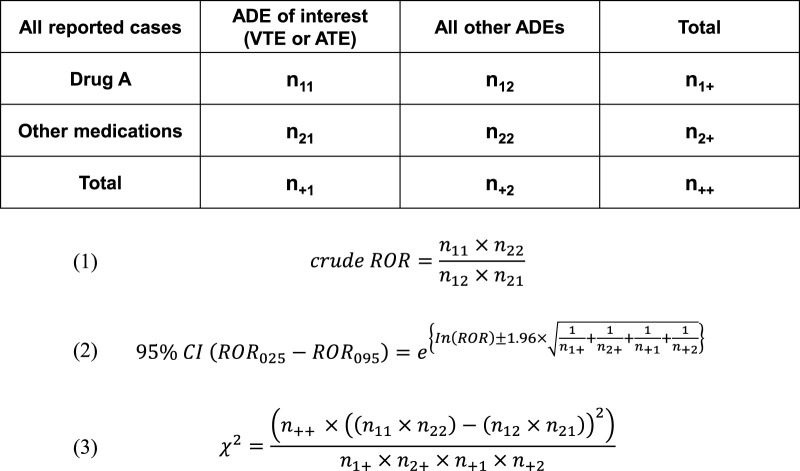
Calculation of cRORs, 95% CIs, and χ^2^ values for VTE and ATE reports as adverse drug events associated with drugs including female hormones, male hormones, and analgesics, based on 2 × 2 contingency tables. ADE: Adverse Drug Event.

**FIGURE 3:**
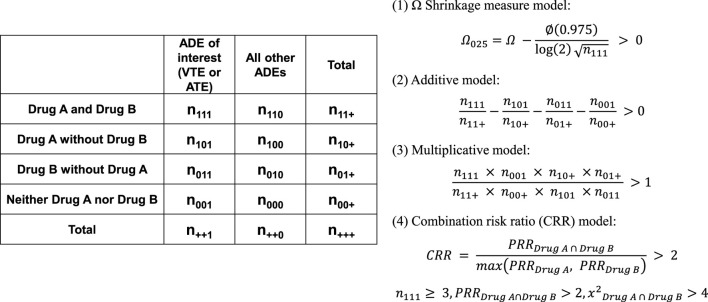
DDI analysis for VTE and ATE based on the number of reported cases involving female or male hormones (Drug A) and analgesics (Drug B). Analyses are conducted using a 4 × 2 contingency table. ADE, Adverse Drug Event.

For each instance, 2 × 2 contingency tables were created based on the presence or absence of drugs, including sex hormones (female and male), analgesics (NSAIDs and acetaminophen), and the adverse events (VTE and ATE). A total of 26 sex hormones (12 male hormones and 14 female hormones) and 45 analgesics (including NSAIDs and acetaminophen) were comprehensively evaluated. This analytical framework was intentionally designed to include all agents classified within these two therapeutic categories. Crude RORs (cRORs), 95% confidence intervals (95%CIs), and chi-squared (χ^2^) values were calculated using Equations 1–3 presented in Figure 2:

Subsequently, for all reported cases, 4 × 2 contingency tables were constructed to assess the presence or absence of VTE and ATE during concomitant use of drugs A and B. DDIs were then evaluated using 4 × 2 contingency tables, applying four statistical methods to assess the impact of two-drug combinations: (1) Ω shrinkage measure model (Norén et al., 2006; 2008), (2) additive model, (3) multiplicative model (Thakrar et al., 2007), and (4) CRR model (Susuta and Takahashi, 2014) (Figure 3). Concomitant drug use was defined as the registration of drugs under the same primary ID.

## 3 Result

The FAERS database included 15,957,559 unduplicated case reports, among which 162,846 were related to VTE and 283,197 to ATE.

First, a disproportionality analysis focusing on individual drugs was performed for VTE. cRORs, 95% confidence intervals (CIs; lower bound of the 95% CI > 1.0), and χ^2^ statistics for drugs showing significant associations with VTE are provided in the Supplementary Material (Supplementary Table S4). These included 12 female hormones, 7 testosterone preparations categorized as male hormones, and 22 analgesics (NSAIDs and acetaminophen).

Similarly, a disproportionality analysis focusing on individual drugs was conducted for ATE. The cRORs, 95% CIs (lower bound of the 95% CI > 1.0), and χ^2^ statistics for significantly associated drugs are summarized in the Supplementary Material (Supplementary Table S5), comprising 4 female hormones, 7 testosterone preparations as male hormones, and 16 analgesics (NSAIDs and acetaminophen).

### 3.1 Drug–drug interaction analysis for VTE between sex hormones and analgesics

DDI analysis was performed using four algorithms on 4 × 2 contingency tables: the Ω shrinkage measure model, additive model, multiplicative model, and CRR model.

The DDI results that met the criteria of *n*_111_ ≥ 50 and the presence of positive signals in at least three of the four analytical models are summarized in Table 1. Drospirenone, ethinylestradiol, norelgestromin, desogestrel, medroxyprogesterone, and levonorgestrel showed a tendency toward consistent positive signals (defined as the number of positive signals in the four models ≥3) across multiple algorithms. Notably, the following drug pairs showed elevated interaction coefficients across models: drospirenone with ketorolac (Ω_025_ = 0.97; additive model = 0.34; multiplicative model = 1.62; CRR model = 2.02), drospirenone with nabumetone (Ω_025_ = 0.78; additive model = 0.34; multiplicative model = 2.5; CRR model = 2.01), ethinylestradiol with ketorolac (1.36 [Ω_025_], 0.29 [additive], 2.09 [multiplicative], 2.52 [CRR]), ethinylestradiol with piroxicam (1.23 [Ω_025_], 0.27 [additive], 1.82 [multiplicative], 2.43 [CRR]), norelgestromin with acetaminophen (1.20 [Ω_025_], 0.12 [additive], 1.7 [multiplicative], and 2.97 [CRR]), norelgestromin with ibuprofen (1.26 [Ω_025_], 0.15 [additive], 1.7 [multiplicative], and 3.39 [CRR]), desogestrel with naproxen (1.23 [Ω_025_], 0.13 [additive], 1.95 [multiplicative], and 2.47 [CRR]), desogestrel with celecoxib (2.05 [Ω_025_], 0.31 [additive], 2.77 [multiplicative], and 4.52 [CRR]), desogestrel with meloxicam (2.39 [Ω_025_], 0.39 [additive], 5.4 [multiplicative], and 5.37 [CRR]), desogestrel with piroxicam (2.54 [Ω_025_], 0.47 [additive], 4.33 [multiplicative], and 6.44 [CRR]), medroxyprogesterone with ibuprofen (0.5 [Ω_025_], 0.03 [additive], 1.18 [multiplicative], and 2.3 [CRR]), levonorgestrel with acetaminophen (0.84 [Ω_025_], 0.02 [additive], 2.14 [multiplicative], and 2.02 [CRR]), levonorgestrel with ibuprofen (0.90 [Ω_025_], 0.03 [additive], 2.31 [multiplicative], and 2.17 [CRR]), respectively.

**TABLE 1:** DDI analysis of VTE reporting between female hormones and analgesics. Drug pairs with n_111_ ≥ 50 and positive signals in ≥3 of the four models are shown.

Female hormone vs. analgesics	n_111_	n_11+_	E_111_	Ω Shrinkage model Ω (Ω_025_–Ω_975_)		Additive model		Multiplicative model		CRR model	
Drospirenone vs.											
Acetaminophen	3,311	6,694	1,665.0	0.99 (0.94–1.04)	+	0.24	+	1.31	+	1.63	−
Ibuprofen	2,485	4,983	1,288.6	0.95 (0.89–1.00)	+	0.24	+	1.63	+	1.64	−
Naproxen	950	1,944	540.3	0.81 (0.72–0.91)	+	0.21	+	1.67	+	1.59	−
Ketorolac	354	569	162.2	1.12 (0.97–1.27)	+	0.34	+	1.62	+	2.02	+
Meloxicam	154	329	94.7	0.70 (0.47–0.93)	+	0.18	+	1.29	+	1.52	−
Diclofenac	152	348	100.3	0.60 (0.37–0.83)	+	0.15	+	1.19	+	1.42	−
Nabumetone	77	124	35.6	1.10 (0.78–1.42)	+	0.34	+	2.5	+	2.01	+
Indometacin	61	109	31.4	0.95 (0.59–1.31)	+	0.27	+	1.73	+	1.81	−
Etodolac	57	95	27.4	1.04 (0.67–1.42)	+	0.31	+	1.75	+	1.94	−
Ethinylestradiol vs.											
Acetaminophen	4,162	16,179	2,348.6	0.83 (0.78–0.87)	+	0.11	+	1.17	+	1.49	−
Ibuprofen	3,119	11,217	1,618.4	0.95 (0.90–1.00)	+	0.13	+	1.93	+	1.6	−
Naproxen	1,259	4,453	671.9	0.91 (0.83–0.99)	+	0.13	+	2.05	+	1.61	−
Ketorolac	365	818	127.6	1.51 (1.36–1.66)	+	0.29	+	2.09	+	2.52	+
Meloxicam	351	1,433	221.3	0.66 (0.51–0.82)	+	0.09	+	1.54	+	1.39	−
Piroxicam	190	442	69.7	1.44 (1.23–1.64)	+	0.27	+	1.82	+	2.43	+
Nabumetone	80	250	38.7	1.04 (0.72–1.35)	+	0.17	+	2.3	+	1.81	−
Etodolac	69	195	30.4	1.17 (0.83–1.51)	+	0.2	+	2.05	+	2	−
Indometacin	67	324	50.6	0.40 (0.06–0.75)	+	0.05	+	1.14	+	1.17	−
Norelgestromin vs.											
Acetaminophen	110	573	39.4	1.47 (1.20–1.74)	+	0.12	+	1.7	+	2.97	+
Ibuprofen	73	334	24.0	1.59 (1.26–1.92)	+	0.15	+	1.7	+	3.39	+
Desogestrel vs.											
Diclofenac	313	1,906	134.9	1.21 (1.05–1.37)	+	0.09	+	2.13	+	1.91	−
Naproxen	198	932	73.1	1.43 (1.23–1.63)	+	0.13	+	1.95	+	2.47	+
Celecoxib	188	483	38.9	2.26 (2.05–2.46)	+	0.31	+	2.77	+	4.52	+
Meloxicam	167	361	27.0	2.61 (2.39–2.83)	+	0.39	+	5.4	+	5.37	+
Piroxicam	158	285	22.9	2.76 (2.54–2.99)	+	0.47	+	4.33	+	6.44	+
Medroxyprogesterone vs.											
Ibuprofen	93	1,478	53.5	0.79 (0.50–1.08)	+	0.03	+	1.18	+	2.3	+
Levonorgestrel vs.											
Acetaminophen	143	3,796	67.5	1.08 (0.84–1.31)	+	0.02	+	2.14	+	2.02	+
Ibuprofen	122	2,649	54.5	1.15 (0.90–1.41)	+	0.03	+	2.31	+	2.17	+

n_111_, number of VTE, events where the female hormone and the analgesic are used concomitantly; n_11+_, number of total adverse events where the female hormone and the analgesic were used concomitantly; E_111_, expected value in the Ω shrinkage measure model; +, positive signal; -, non-positive signal.

From the perspective of analgesics, ibuprofen produced positive signals when combined with five female hormones—drospirenone, ethinylestradiol, norelgestromin, medroxyprogesterone, and levonorgestrel—in three algorithms (Ω shrinkage measure, additive, and multiplicative models). Similarly, acetaminophen produced positive signals when combined with four female hormones drospirenone, ethinylestradiol, norelgestromin, and levonorgestrel) according to the same three DDI algorithms.

Table 2 presents the results of the DDI analyses between male hormones and analgesics that were frequently co-reported in VTE cases (drug pairs with n_111_ ≥ 10 are shown). Only methyltestosterone with acetaminophen generated positive signal in one DDI detection algorithm (1.01 [multiplicative]), whereas other combinations showed no consistent positive signals.

**TABLE 2:** DDI analysis of VTE reporting between male hormones and analgesics. Drug pairs with n_111_ ≥ 10 are shown.

Male hormone vs. Analgesics	n_111_	n_11+_	E_111_	Ω Shrinkage model Ω (Ω_025_–Ω_975_)		Additive model		Multiplicative model		CRR model	
Testosterone vs.											
Acetaminophen	159	3,765	342.1	−1.1 (−1.33 to −0.88)	−	−0.05	−	0.27	−	0.52	−
Celecoxib	32	708	64.1	−0.99 (−1.49 to −0.49)	−	−0.05	−	0.26	−	0.55	−
Ibuprofen	32	1,161	106.0	−1.71 (−2.21 to −1.21)	−	−0.07	−	0.16	−	0.34	−
Meloxicam	23	631	53.4	−1.2 (−1.79 to −0.61)	−	−0.05	−	0.32	−	0.44	−
Naproxen	22	763	65.9	−1.56 (−2.16 to −0.96)	−	−0.06	−	0.22	−	0.35	−
Diclofenac	15	671	56.3	−1.87 (−2.60 to −1.14)	−	−0.06	−	0.21	−	0.27	−
Testosterone cipionate vs.											
Acetaminophen	51	1,139	149.8	−1.54 (−1.94 to −1.15)	−	−0.09	−	0.19	−	0.39	−
Testosterone enantate vs.											
Acetaminophen	28	290	32.4	−0.21 (−0.74–0.33)	−	−0.02	−	0.5	−	0.92	−
Methyltestosterone vs.											
Acetaminophen	11	520	10.2	0.11 (−0.74–0.96)	−	0	−	1.01	+	1.13	−

n_111_, number of VTE, events where the male hormone and analgesic are used concomitantly; n_11+_, number of total adverse events where the male hormone and analgesic are used concomitantly; E_111_, expected value in the Ω shrinkage measure model; +, positive signal; −, non-positive signal.

### 3.2 Drug–drug interaction analysis for ATE between sex hormones and analgesics

The analysis of DDIs related to ATE reports was performed using 4 × 2 contingency tables to assess the impact of concurrent use of female and male hormones with various analgesics. Similarly to the DDI analysis for VTE, female hormones, a tendency toward consistent positive signals across algorithms was also observed. The DDI results that met the criteria of *n*_111_ ≥ 50 and the presence of positive signals in at least three of the four analytical models are summarized in Table 3. The following drug pairs showed elevated interaction coefficients across models: Medroxyprogesterone with acetaminophen (Ω_025_ = 0.83; additive model = 0.02; multiplicative model = 2.14; CRR model = 2.05), among three female hormones combined with representative analgesics frequently co-reported in ATE cases, Estrogen showed positive signals when concomitantly used with acetaminophen (0.79 [Ω_025_], 0.03 [additive], and 1.78 [multiplicative]), ibuprofen (0.36 [Ω_025_], 0.02 [additive], and 1.75 [multiplicative]), naproxen (0.09 [Ω_025_], 0.01 [additive], and 1.27 [multiplicative]), diclofenac (0.08 [Ω_025_], 0.01 [additive], and 1.36 [multiplicative]), drospirenone used with acetaminophen (0.35 [Ω_025_], 1.35 [additive], and 1.45 [multiplicative]), ibuprofen (0.50 [Ω_025_], 0.02 [additive], and 1.86 [multiplicative]), naproxen (0.20 [Ω_025_], 0.01 [additive], and 1.38 [multiplicative]), medroxyprogesterone used with ibuprofen (0.60 [Ω_025_], 0.02 [additive], and 2.22 [multiplicative]) and ethinylestradiol used with ibuprofen (0.31 [Ω_025_], 0.01 [additive], and 1.66 [multiplicative]), naproxen (0.15 [Ω_025_], 0.01 [additive], and 1.35 [multiplicative]) across the three DDI algorithms.

**TABLE 3:** DDI analysis of ATE reporting between female hormones and analgesics. Drug pairs with n_111_ ≥ 50 and positive signals in ≥3 of the four models are shown.

Female hormone vs. analgesics	n_111_	n_11+_	E_111_	Ω Shrinkage model Ω (Ω_025_–Ω_975_)	Additive model			Multiplicative model		CRR model	
Estrogen vs.											
Acetaminophen	519	8,918	274.7	0.92 (0.79–1.04)	+	0.03	+	1.78	+	1.82	−
Ibuprofen	110	2,255	70.8	0.63 (0.36–0.90)	+	0.02	+	1.75	+	1.52	−
Naproxen	80	1,818	60.4	0.40 (0.09–0.72)	+	0.01	+	1.27	+	1.37	−
Diclofenac	60	1,375	43.9	0.45 (0.08–0.81)	+	0.01	+	1.36	+	1.36	−
Drospirenone vs.											
Acetaminophen	287	6,694	201.1	0.51 (0.35–0.68)	+	0.01	+	1.35	+	1.45	−
Ibuprofen	221	4,983	136.6	0.69 (0.50–0.88)	+	0.02	+	1.86	+	1.5	−
Naproxen	84	1,944	59.1	0.50 (0.20–0.81)	+	0.01	+	1.38	+	1.46	−
Medroxyprogesterone vs.											
Acetaminophen	164	3,778	78.9	1.05 (0.83–1.27)	+	0.02	+	2.14	+	2.05	+
Ibuprofen	55	1,478	27.7	0.98 (0.60–1.36)	+	0.02	+	2.22	+	1.92	−
Ethinylestradiol vs.											
Ibuprofen	278	11,217	199.5	0.48 (0.31–0.65)	+	0.01	+	1.66	+	1.4	−
Naproxen	115	4,453	86.4	0.41 (0.15–0.67)	+	0.01	+	1.35	+	1.32	−

n_111_, number of ATE, events where female hormone and analgesic are used concomitantly; n_11+_, number of total adverse events where female hormone and analgesic are used concomitantly; E_111_, expected value in the Ω shrinkage measure model; +, positive signal; -, non-positive signal.

Table 4 presents the results of the DDI analyses between male hormones and analgesics for ATE (n_111_ ≥ 10). Only methyltestosterone with acetaminophen generated positive signals in two DDI algorithms (0.01 [additive], 1.25 [multiplicative]), whereas other combinations showed no positive signals.

**TABLE 4:** DDI analysis of ATE reporting between male hormones and analgesics. Drug pairs with **n**_111_ ≥ 10 are shown.

Male hormone vs. analgesics	n_111_	n_11+_	E_111_	Ω Shrinkage model Ω (Ω_025_–Ω_975_)		Additive model		Multiplicative model		CRR model	
Testosterone vs.											
Acetaminophen	262	3,765	468	−0.84 (−1.01 to −0.66)	−	−0.06	−	0.48	−	0.58	−
Ibuprofen	68	1,161	138	−1.01 (−1.36 to −0.67)	−	−0.06	−	0.55	−	0.49	−
Naproxen	51	763	91	−0.83 (−1.23 to −0.44)	−	−0.05	−	0.51	−	0.56	−
Celecoxib	47	708	116	−1.29 (−1.7 to −0.88)	−	−0.11	−	0.13	−	0.56	−
Meloxicam	43	631	75	−0.79 (−1.22 to −0.36)	−	−0.05	−	0.56	−	0.57	−
Diclofenac	40	671	79.5	−0.98 (−1.43 to −0.54)	−	−0.06	−	0.49	−	0.5	−
Rofecoxib	17	66	30.1	−0.81 (−1.49 to −0.12)	−	−0.26	−	0.09	−	0.58	−
Indometacin	10	124	16.9	−0.73 (−1.62–0.17)	−	−0.06	−	0.3	−	0.68	−
Testosterone cipionate vs.											
Acetaminophen	83	1,139	223	−1.42 (−1.73 to −1.11)	−	−0.12	−	0.31	−	0.41	−
Ibuprofen	18	333	60.5	−1.72 (−2.39 to −1.06)	−	−0.13	−	0.33	−	0.3	−
Diclofenac	16	182	32.5	−1 (−1.71 to −0.3)	−	−0.09	−	0.48	−	0.5	−
Meloxicam	13	212	38.2	−1.52 (−2.3 to −0.73)	−	−0.12	−	0.33	−	0.35	−
Naproxen	11	194	35.1	−1.63 (−2.48 to −0.78)	−	−0.12	−	0.29	−	0.32	−
Methyltestosterone vs.											
Rofecoxib	40	110	46.5	−0.21 (−0.66–0.23)	−	−0.06	−	0.57	−	0.82	−
Acetaminophen	27	520	19.8	0.44 (−0.11–0.98)	−	0.01	+	1.25	+	1.38	−
Testosterone enantate vs.											
Acetaminophen	14	290	31.3	−1.13 (−1.89 to −0.38)	−	−0.06	−	0.38	−	0.49	−

n_111_, number of ATE, events where male hormone and analgesic are used concomitantly; n_11+_, number of total adverse events where male hormone and analgesic are used concomitantly; E_111_, expected value in the Ω shrinkage measure model; +, positive signal; -, non-positive signal.

Table 5 shows drug pairs of sex hormones and analgesics that demonstrated positive signals across all four analytical models in the DDI analysis. The results indicate that, in VTE cases, six female hormones—drospirenone, ethinylestradiol, norelgestromin, desogestrel, medroxyprogesterone, and levonorgestrel—demonstrated positive signals in combination with analgesics across the four analytical algorithms. In contrast, in ATE cases, consistency across algorithms was observed only for medroxyprogesterone and acetaminophen. By comparison, no consistent DDI signals across the four analytical algorithms were identified for male hormones in either VTE or ATE cases. A heat map of the four DDI indices for both VTE and ATE are provided in Supplementary Table S6.

**TABLE 5:** DDI analysis of sex hormones and analgesics with positive signals across all four models.

Female hormone vs. analgesics for VTE	n_111_	n_11+_	E_111_	Ω Shrinkage model Ω (Ω_025_ – Ω_975_)		Additive model		Multiplicative model		CRR model	
Drospirenone vs.											
Ketorolac	354	569	162.2	1.12 (0.97 – 1.27)	+	0.34	+	1.62	+	2.02	+
Nabumetone	77	124	35.6	1.10 (0.78 – 1.42)	+	0.34	+	2.5	+	2.01	+
Ethinylestradiol vs.											
Ketorolac	365	818	127.6	1.51 (1.36 – 1.66)	+	0.29	+	2.09	+	2.52	+
Piroxicam	190	442	69.7	1.44 (1.23 – 1.64)	+	0.27	+	1.82	+	2.43	+
Norelgestromin vs.											
Acetaminophen	110	573	39.4	1.47 (1.20 – 1.74)	+	0.12	+	1.7	+	2.97	+
Ibuprofen	73	334	24.0	1.59 (1.26 – 1.92)	+	0.15	+	1.7	+	3.39	+
Desogestrel vs.											
Naproxen	198	932	73.1	1.43 (1.23 – 1.63)	+	0.13	+	1.95	+	2.47	+
Celecoxib	188	483	38.9	2.26 (2.05 – 2.46)	+	0.31	+	2.77	+	4.52	+
Meloxicam	167	361	27.0	2.61 (2.39 – 2.83)	+	0.39	+	5.4	+	5.37	+
Piroxicam	158	285	22.9	2.76 (2.54 – 2.99)	+	0.47	+	4.33	+	6.44	+
Medroxyprogesterone vs.											
Ibuprofen	93	1,478	53.5	0.79 (0.50 – 1.08)	+	0.03	+	1.18	+	2.3	+
Levonorgestrel vs.											
Acetaminophen	143	3,796	67.5	1.08 (0.84 – 1.31)	+	0.02	+	2.14	+	2.02	+
Ibuprofen	122	2,649	54.5	1.15 (0.90 – 1.41)	+	0.03	+	2.31	+	2.17	+

Female hormone vs. analgesics for ATE	n_111_	n_11+_	E_111_	Ω Shrinkage model Ω (Ω_025_ – Ω_975_)	​	Additive model		Multiplicative model		CRR model	
Medroxyprogesterone vs.											
Acetaminophen	164	3,778	78.9	1.05 (0.83 – 1.27)	+	0.02	+	2.14	+	2.05	+

n_111_, number of VTE, or ATE, events where female hormone and analgesic are used concomitantly; n_11+_, number of total adverse events where female hormone and analgesic are used concomitantly; E_111_, expected value in the Ω shrinkage measure model; +, positive signal; −, non-positive signal.

## 4 Discussion

Due to limited data on how DDIs affect thrombotic events, we conducted a broad exploratory study using the FAERS database to examine DDIs related to VTE and ATE reports associated with the combined use of hormones—both male and female—and analgesics. The disproportionality analysis revealed that several types of female and male hormones, as well as analgesics, were associated with higher rates of VTE and ATE reporting. Moreover, use of female hormones together with analgesics correlated with an increase in reported thrombotic events, particularly VTE. Fewer drug pairs showed consistent positive signals for ATE than for VTE. In contrast, concomitant use of male hormones with analgesics did not yield consistent positive DDI signals related to VTE or ATE.

Despite several proposed methods, no standard exists for detecting DDI signals in SRSs. This study employed four DDI algorithms based on 4 × 2 contingency tables (Noguchi et al., 2019; 2021). The Ω shrinkage measure model is considered conservative and effective at reducing sensitivity to random fluctuations in disproportionality measures based on rare cases (Norén et al., 2006; 2008; Noguchi et al., 2019). Additive and multiplicative models tend to be more sensitive but may yield false associations due to noise and bias. Confirming signals with multiple statistical models is a reasonable approach (Honma et al., 2024; Ji et al., 2025). Crude RORs can be influenced by various confounders and thus should not be interpreted as risk indicators. Accordingly, cRORs were calculated in the current study for reference. Logistic regression can provide adjusted RORs, but this approach is complex for multiple drug combinations, and such RORs derived from different logistic models are not directly comparable. Thus, four DDI algorithms using 4 × 2 contingency tables were employed to assess DDIs between sex hormones and analgesics.

Estrogen and progesterone are commonly used as contraceptives and are widely recognized as risk factors for VTE, DVT, and PE (Practice Committee of the American Society for Reproductive Medicine. Electronic address: ASRM@asrm.org and Practice Committee of the American Society for Reproductive Medicine, 2017). Estrogen promotes VTE by increasing coagulation factors, including fibrinogen, prothrombin, and factors VII, VIII, and X, while decreasing anticoagulant factors like antithrombin, tissue factor pathway inhibitor (TFPI), and protein S. This shift in the hemostatic balance favors blood clot formation and induces resistance to activated protein C (APC), increasing the risk of VTE. Notably, lower levels of protein S and TFPI are associated with increased APC resistance, a key mechanism underlying the heightened VTE risk (Scarabin et al., 1997; Oger et al., 2003; Tchaikovski and Rosing, 2010). This risk varies with the estrogen dose and the type of progesterone; even at the same estrogen dose, differences in progesterone type can influence VTE risk (Oedingen et al., 2018). The interaction between estrogen and progesterone is considered a key determinant of their impact on the coagulation system.

Testosterone replacement therapy (TRT) is indicated for men with clinical symptoms of hypogonadism and consistently low serum testosterone levels. TRT has been evaluated for potential thrombotic risks from both ATE and VTE perspectives (Finkle et al., 2014; Martinez et al., 2016). Several mechanisms may explain testosterone-associated thrombosis, including erythrocytosis mediated by erythropoietin stimulation and hepcidin suppression, as well as enhanced platelet aggregation via thromboxane A2 receptor upregulation (Matsuda et al., 1994; Ajayi et al., 1995; Bachman et al., 2010; 2014). From a regulatory standpoint, in 2014 the FDA required a class-wide labeling update to include a general warning about VTE based on postmarket reports of venous blood clots unrelated to polycythemia (FDA Drug Safety Communications, 2014; FDA Drug Safety Communication, 2015b). However, in a recent double-blind, placebo-controlled trial (TRAVERSE), TRT was not associated with an excess incidence of major adverse cardiovascular events compared with placebo, still, PE occurred more frequently in the TRT group as a safety finding (Lincoff et al., 2023). Consistent with this, a meta-analysis in men with low baseline testosterone did not demonstrate a statistically significant increase in ATE with TRT, whereas DVT risk remains uncertain (Cannarella et al., 2024). In 2025, following review of TRAVERSE and postmarketing studies, the FDA issued class-wide labeling changes that removed boxed-warning language related to increased risk of adverse cardiovascular outcomes and added warnings regarding increased blood pressure (FDA issues, 2025). In our analysis, no consistent DDI signals were detected between male hormones and analgesics. This may suggest that male hormones with relatively low intrinsic thrombotic risk do not substantially contribute to increased thrombotic events when co-administered with analgesics.

NSAIDs are commonly used to relieve pain and reduce inflammation by blocking cyclooxygenase type 1 (COX-1) or type 2 (COX-2), which produce prostaglandins, the mediators of pain. However, NSAID use may also be associated with an increased risk of VTE and ATE (Chan et al., 2006; Ungprasert et al., 2015; Lee et al., 2016). In particular, high doses of NSAIDs have been linked to an increased risk of ATE, leading to the removal of rofecoxib and valdecoxib from the market. In 2005, the FDA issued a warning regarding the cardiovascular risks of NSAIDs (FDA Safety and Availability, 2005), and in 2015 strengthened the boxed warning on all prescriptions and over-the-counter NSAID products to emphasize the increased risk of myocardial infarction and stroke. Currently, all NSAIDs (excluding low-dose aspirin) are considered to pose cardiovascular risks, and high-dose, long-term use should be avoided. When necessary, the lowest effective dose for the shortest duration is recommended (FDA Drug Safety Communication, 2015a). Due to the difficulty in interpreting aspirin dose, aspirin was excluded from the current analyses.

NSAIDs are thought to promote thrombogenesis through multiple pharmacological mechanisms. Notably, COX-2 selective agents inhibit COX-2 in the vascular endothelium, reducing the production of prostacyclin (PGI), which possesses anti-thrombotic properties. This disruption in the PGI_2_/TXA_2_ equilibrium promotes platelet aggregation and increases thrombus formation. Additionally, NSAID-induced platelet COX-1 inhibition is transient, permitting intermittent platelet activation and subsequent release of coagulation factors and inflammatory mediators, thereby enhancing tissue factor expression and thrombin generation, which further elevates thrombosis risk (Cheng et al., 2002; Radi and Khan, 2019).

Regarding drug combinations, differences in the biological mechanisms underlying thrombosis may partly explain the observed patterns of DDI signals. The stronger signals observed with female hormones may reflect greater mechanistic convergence with NSAID-related pathways. In contrast, as thrombosis associated with male hormone therapy has been linked to erythrocytosis and increased blood viscosity Guo et al. (2015) the degree of overlap with NSAID-related pathways may be more limited.

Several NSAIDs, including piroxicam and ketorolac, when combined with female hormones, showed consistent positive signals for VTE across multiple algorithms. NSAIDs differ substantially in their chemical structures, elimination half-lives, and selectivity for COX-1 and COX-2, all of which may influence thrombotic risk. Piroxicam has a long elimination half-life of about 50 h, whereas ketorolac is frequently administered intravenously, potentially resulting in differences in systemic exposure and acute pharmacodynamic effects. Given that the actual degree of COX inhibition is known to vary in a dose- and concentration-dependent manner, interpretation of these findings requires consideration of clinically relevant dosing regimens and drug exposure parameters. In addition, accumulating evidence indicates that individual NSAIDs possess pharmacological properties beyond cyclooxygenase (COX) inhibition, including differences in COX-1/COX-2 selectivity and engagement of additional off-target pathways (Oprea et al., 2015; Roggero et al., 2025). Although direct mechanistic evidence remains limited, such pharmacological heterogeneity among NSAIDs may partly contribute to differences in thrombogenic potential when co-administered with hormone therapies.

The DDI analysis in the current study showed that concomitant use of female hormones and analgesics was associated with increased reporting rates of VTE, consistent with findings from a nationwide Danish cohort study (Meaidi et al., 2023). Among women aged 19–49 years in Denmark, those using oral contraceptives classified as high risk for VTE showed a more pronounced increase in VTE risk following concomitant use of NSAIDs, compared with women not using hormonal contraceptives. High-risk oral contraceptives, defined based on formulation and ethinylestradiol content, include desogestrel-, drospirenone-, and norelgestromine-containing products. The observed significant increase in VTE risk with concomitant NSAID use for these contraceptives closely parallels the findings of the present study. Although the cohort study and the FAERS database differ substantially in data characteristics and collection methods, the concordant direction of the findings provides important external validity supporting our results.

In contrast to female hormones, concomitant administration of male hormones and NSAIDs did not lead to increased VTE or ATE reporting. Moreover, no previous epidemiological studies were identified that specifically examine the impact of concomitant drug use on ATE, such as the combinations involving female hormones with NSAIDs or acetaminophen, or male hormones with these analgesics. This lack of evidence appears consistent with the limited or absent reproducible DDI signals in these contexts.

This study has several limitations. First, the pairwise DDIs analysis in this study was limited to the evaluation of interactions between two drugs; therefore, the effects of triple drug combinations could not be assessed. Estrogen and progesterone are frequently used in combination as oral contraceptives. Our investigation using the FAERS showed that a substantial proportion of progesterone-related cases involved concomitant ethinylestradiol use (see Supplementary Table S7 for details). Specifically, norelgestromin, drospirenone, norgestrel, etonogestrel, and desogestrel exhibited high rates of concomitant use (>85%) with ethinylestradiol. Therefore, the increased reporting of thromboembolic events linked to these progesterone preparations may be attributable to the triple concomitant use of ethinylestradiol and NSAIDs. Although logistic regression analysis could be feasible to analyze triple combinations, constructing a stable multivariable model becomes challenging because of the large number of hormones and analgesics and their possible combinations. In addition, the uneven distribution of concomitant drug use across drug pairs due to fixed combinations may introduce multicollinearity among covariates. Further studies are required to clarify the potential impact of more complex drug combinations.

As the second limitation, because this analysis was based on the SRS, it is inherently subject to reporting bias and confounding. Adverse events may be either under-reported or over-reported, and, as illustrated by the examples of rofecoxib and testosterone, external factors such as media attention and regulatory actions may influence reporting behavior. These issues should be taken into consideration when interpreting the results. Furthermore, the lack of reliable data on drug user populations (i.e., denominator for incidence calculations) prevents accurate estimation of event frequency. Moreover, limited case-level details hinder the complete control of confounding factors such as patient history, including preexisting conditions (e.g., cancer or atherosclerosis) and lifestyle attributes (e.g., age, obesity, or smoking status) (Michel et al., 2017). Especially, cancer is a well-established risk factor for thrombosis and NSAIDs are frequently used for cancer-related pain control. To address this potential confounding, we conducted separate DDI analyses restricted to patients without cancer-related indications. In addition, age-stratified analyses were performed in younger (20–59 years) and older (60–100 years) populations. As a result, a similar tendency was observed across these subgroup analyses (data not shown). These results suggest that the observed sex-specific interaction patterns were not solely driven by cancer-related confounding or age distribution. As an additional limitation, our definition of concomitant use may have included cases in which drugs were switched or used sequentially within the reporting period. Importantly, studies relying solely on the SRS cannot establish causality. Thus, although findings aligned with previous epidemiological evidence and support conclusions drawn from FAERS analyses, newly identified signals should be interpreted as hypotheses. Robust causality assessment will require further validation via prospective cohort studies, mechanistic investigations, and real-world data analyses to supplement these findings.

## 5 Conclusion

To our knowledge, this study is the first to employ FAERS data to evaluate the impact of concomitant use of sex hormones and analgesics on thromboembolic events. The present study suggests that combinations of drospirenone- or desogestrel-containing oral contraceptives with ethinylestradiol and specific NSAIDs (ketorolac, piroxicam, or meloxicam) may warrant continued pharmacovigilance and careful post-marketing surveillance. The analysis also revealed differences in how concomitant analgesic use affects thrombotic event reporting for male versus female hormones. Notably, the increased reports of VTE associated with the combined use of female hormones and analgesics align with prior epidemiological findings, supporting the utility of FAERS-based DDI analysis. Furthermore, these results underscore the complementary role of this analytical approach in identifying potential DDIs that may be overlooked by epidemiological studies.

## Data Availability

Publicly available datasets were analyzed in this study. This data can be found here: https://www.fda.gov/drugs/drug-approvals-and-databases/fda-adverse-event-reporting-system-faers-database.
