# Supplementary material for: Drug-drug interactions between sex hormones and analgesics linked to thromboembolic events: an FDA adverse event reporting system analysis
Source: Front Pharmacol. 2026 Apr 21;17:1791198. doi: 10.3389/fphar.2026.1791198 (PMC13139130; doi:10.3389/fphar.2026.1791198)
Supplement: Supplementary file 1 [file Supplementaryfile1.docx]

# S1: Preferred Terms for the Definition of Embolic and Thrombotic Events, Venous (SMQ 20000084)

| PT_CODE | PT_NAME |
| --- | --- |
| 10084527 | Aseptic cavernous sinus thrombosis |
| 10003880 | Axillary vein thrombosis |
| 10076837 | Brachiocephalic vein occlusion |
| 10063363 | Brachiocephalic vein thrombosis |
| 10006537 | Budd-Chiari syndrome |
| 10052698 | Catheterisation venous |
| 10007830 | Cavernous sinus thrombosis |
| 10053377 | Central venous catheterisation |
| 10083037 | Cerebral venous sinus thrombosis |
| 10008138 | Cerebral venous thrombosis |
| 10079209 | Compression garment application |
| 10051055 | Deep vein thrombosis |
| 10066881 | Deep vein thrombosis postoperative |
| 10014522 | Embolism venous |
| 10078810 | Hepatic vein embolism |
| 10058991 | Hepatic vein occlusion |
| 10019713 | Hepatic vein thrombosis |
| 10051031 | Homans' sign positive |
| 10058992 | Iliac vein occlusion |
| 10070911 | Inferior vena cava syndrome |
| 10058987 | Inferior vena caval occlusion |
| 10081850 | Jugular vein embolism |
| 10076835 | Jugular vein occlusion |
| 10023237 | Jugular vein thrombosis |
| 10075428 | Mahler sign |
| 10069727 | May-Thurner syndrome |
| 10087917 | Mesenteric vein embolism |
| 10027402 | Mesenteric vein thrombosis |
| 10027403 | Mesenteric venous occlusion |
| 10029925 | Obstetrical pulmonary embolism |
| 10073708 | Obstructive shock |
| 10074349 | Ophthalmic vein thrombosis |
| 10072059 | Ovarian vein thrombosis |
| 10050216 | Paget-Schroetter syndrome |
| 10034272 | Pelvic venous thrombosis |
| 10034324 | Penile vein thrombosis |
| 10083103 | Peripheral vein occlusion |
| 10087102 | Peripheral vein thrombosis |
| 10082853 | Peripheral vein thrombus extension |
| 10048874 | Phlebectomy |
| 10073979 | Portal vein cavernous transformation |
| 10082030 | Portal vein embolism |
| 10058989 | Portal vein occlusion |
| 10036206 | Portal vein thrombosis |
| 10077623 | Portosplenomesenteric venous thrombosis |
| 10063909 | Post procedural pulmonary embolism |
| 10048591 | Post thrombotic syndrome |
| 10050902 | Postoperative thrombosis |
| 10036300 | Postpartum venous thrombosis |
| 10037377 | Pulmonary embolism |
| 10037410 | Pulmonary infarction |
| 10037421 | Pulmonary microemboli |
| 10037437 | Pulmonary thrombosis |
| 10068690 | Pulmonary vein occlusion |
| 10037458 | Pulmonary veno-occlusive disease |
| 10037459 | Pulmonary venous thrombosis |
| 10038547 | Renal vein embolism |
| 10056293 | Renal vein occlusion |
| 10038548 | Renal vein thrombosis |
| 10038907 | Retinal vein occlusion |
| 10038908 | Retinal vein thrombosis |
| 10068479 | SI QIII TIII pattern |
| 10087208 | Sigmoid sinus thrombosis |
| 10087968 | Spermatic vein thrombosis |
| 10068122 | Splenic vein occlusion |
| 10041659 | Splenic vein thrombosis |
| 10089094 | Subclavian vein embolism |
| 10079164 | Subclavian vein occlusion |
| 10049446 | Subclavian vein thrombosis |
| 10086210 | Superficial vein thrombosis |
| 10042567 | Superior sagittal sinus thrombosis |
| 10058988 | Superior vena cava occlusion |
| 10042569 | Superior vena cava syndrome |
| 10043570 | Thrombophlebitis |
| 10043581 | Thrombophlebitis migrans |
| 10043586 | Thrombophlebitis neonatal |
| 10043605 | Thrombosed varicose vein |
| 10067270 | Thrombosis corpora cavernosa |
| 10044457 | Transverse sinus thrombosis |
| 10047193 | Vena cava embolism |
| 10048932 | Vena cava filter insertion |
| 10074397 | Vena cava filter removal |
| 10047195 | Vena cava thrombosis |
| 10047209 | Venogram abnormal |
| 10062173 | Venoocclusive disease |
| 10047216 | Venoocclusive liver disease |
| 10077826 | Venous angioplasty |
| 10058990 | Venous occlusion |
| 10062175 | Venous operation |
| 10068605 | Venous recanalisation |
| 10052964 | Venous repair |
| 10063389 | Venous stent insertion |
| 10047249 | Venous thrombosis |
| 10067030 | Venous thrombosis in pregnancy |
| 10061408 | Venous thrombosis limb |
| 10064602 | Venous thrombosis neonatal |
| 10077829 | Visceral venous thrombosis |

# S2: Preferred Terms for the Definition of Embolic and Thrombotic Events, Arterial (SMQ 20000082)

| PT_CODE | PT_NAME |
| --- | --- |
| 10074337 | Acute aortic syndrome |
| 10051592 | Acute coronary syndrome |
| 10000891 | Acute myocardial infarction |
| 10001902 | Amaurosis |
| 10001903 | Amaurosis fugax |
| 10086560 | Aneurysm thrombosis |
| 10002475 | Angioplasty |
| 10087774 | Angiostomy |
| 10087600 | Aortic aneurysm thrombosis |
| 10057617 | Aortic bypass |
| 10002897 | Aortic embolus |
| 10061651 | Aortic surgery |
| 10002910 | Aortic thrombosis |
| 10057794 | Aortogram abnormal |
| 10071026 | Arterectomy |
| 10003140 | Arterectomy with graft replacement |
| 10081731 | Arterial angioplasty |
| 10056418 | Arterial bypass operation |
| 10061655 | Arterial graft |
| 10062599 | Arterial occlusive disease |
| 10087576 | Arterial recanalisation procedure |
| 10084482 | Arterial revascularisation |
| 10061657 | Arterial stent insertion |
| 10052949 | Arterial therapeutic procedure |
| 10003178 | Arterial thrombosis |
| 10061659 | Arteriogram abnormal |
| 10003195 | Arteriogram carotid abnormal |
| 10078636 | Arteriotomy |
| 10063025 | Atherectomy |
| 10076604 | Atherosclerotic plaque rupture |
| 10079735 | Atrial appendage closure |
| 10080843 | Atrial appendage resection |
| 10069020 | Basal ganglia infarction |
| 10048963 | Basilar artery occlusion |
| 10063093 | Basilar artery thrombosis |
| 10005184 | Blindness transient |
| 10069694 | Brachiocephalic artery occlusion |
| 10067744 | Capsular warning syndrome |
| 10071260 | Carotid angioplasty |
| 10007684 | Carotid arterial embolus |
| 10053003 | Carotid artery bypass |
| 10048964 | Carotid artery occlusion |
| 10066102 | Carotid artery stent insertion |
| 10007688 | Carotid artery thrombosis |
| 10007692 | Carotid endarterectomy |
| 10072559 | Carotid revascularisation |
| 10053633 | Cerebellar artery occlusion |
| 10008023 | Cerebellar artery thrombosis |
| 10087440 | Cerebral angioplasty |
| 10008088 | Cerebral artery embolism |
| 10008089 | Cerebral artery occlusion |
| 10081893 | Cerebral artery stent insertion |
| 10008092 | Cerebral artery thrombosis |
| 10089035 | Cerebral bypass surgery |
| 10065384 | Cerebral hypoperfusion |
| 10071508 | Cerebral revascularisation |
| 10058842 | Cerebrovascular insufficiency |
| 10061751 | Cerebrovascular stenosis |
| 10069696 | Coeliac artery occlusion |
| 10050329 | Coronary angioplasty |
| 10052086 | Coronary arterial stent insertion |
| 10011077 | Coronary artery bypass |
| 10011084 | Coronary artery embolism |
| 10011086 | Coronary artery occlusion |
| 10053261 | Coronary artery reocclusion |
| 10011090 | Coronary artery surgery |
| 10011091 | Coronary artery thrombosis |
| 10011101 | Coronary endarterectomy |
| 10049887 | Coronary revascularisation |
| 10075162 | Coronary vascular graft occlusion |
| 10058729 | Embolia cutis medicamentosa |
| 10014513 | Embolism arterial |
| 10014648 | Endarterectomy |
| 10068365 | Femoral artery embolism |
| 10019635 | Hepatic artery embolism |
| 10051991 | Hepatic artery occlusion |
| 10019636 | Hepatic artery thrombosis |
| 10063518 | Hypothenar hammer syndrome |
| 10021338 | Iliac artery embolism |
| 10064601 | Iliac artery occlusion |
| 10088942 | Incomplete atrial appendage closure |
| 10083408 | Internal capsule infarction |
| 10052989 | Intra-aortic balloon placement |
| 10056382 | Intraoperative cerebral artery occlusion |
| 10060840 | Ischaemic cerebral infarction |
| 10061256 | Ischaemic stroke |
| 10051078 | Lacunar infarction |
| 10085044 | Left atrial appendage closure implant |
| 10024242 | Leriche syndrome |
| 10027394 | Mesenteric arterial occlusion |
| 10065560 | Mesenteric arteriosclerosis |
| 10027395 | Mesenteric artery embolism |
| 10027396 | Mesenteric artery stenosis |
| 10071261 | Mesenteric artery stent insertion |
| 10027397 | Mesenteric artery thrombosis |
| 10086596 | Metabolic stroke |
| 10028596 | Myocardial infarction |
| 10028602 | Myocardial necrosis |
| 10086395 | Ophthalmic artery occlusion |
| 10081144 | Ophthalmic artery thrombosis |
| 10033697 | Papillary muscle infarction |
| 10068035 | Penile artery occlusion |
| 10065608 | Percutaneous coronary intervention |
| 10062585 | Peripheral arterial occlusive disease |
| 10069379 | Peripheral arterial reocclusion |
| 10057518 | Peripheral artery angioplasty |
| 10072561 | Peripheral artery bypass |
| 10057525 | Peripheral artery occlusion |
| 10072562 | Peripheral artery stent insertion |
| 10082470 | Peripheral artery surgery |
| 10072564 | Peripheral artery thrombosis |
| 10061340 | Peripheral embolism |
| 10072560 | Peripheral endarterectomy |
| 10071642 | Popliteal artery entrapment syndrome |
| 10066592 | Post procedural myocardial infarction |
| 10058144 | Postinfarction angina |
| 10085250 | Precerebral artery embolism |
| 10036511 | Precerebral artery occlusion |
| 10074717 | Precerebral artery thrombosis |
| 10078867 | Profundaplasty |
| 10085779 | Pseudo-occlusion of internal carotid artery |
| 10087828 | Pulmonary angioplasty |
| 10078201 | Pulmonary artery occlusion |
| 10088551 | Pulmonary artery stent insertion |
| 10063731 | Pulmonary artery therapeutic procedure |
| 10037340 | Pulmonary artery thrombosis |
| 10072893 | Pulmonary endarterectomy |
| 10079988 | Pulmonary tumour thrombotic microangiopathy |
| 10057493 | Renal artery angioplasty |
| 10048988 | Renal artery occlusion |
| 10087816 | Renal artery revascularisation |
| 10038380 | Renal artery thrombosis |
| 10063544 | Renal embolism |
| 10085346 | Renal-limited thrombotic microangiopathy |
| 10038826 | Retinal artery embolism |
| 10038827 | Retinal artery occlusion |
| 10038831 | Retinal artery thrombosis |
| 10086467 | Segmental arterial mediolysis |
| 10049768 | Silent myocardial infarction |
| 10049440 | Spinal artery embolism |
| 10071316 | Spinal artery thrombosis |
| 10074600 | Splenic artery thrombosis |
| 10068677 | Splenic embolism |
| 10066286 | Stress cardiomyopathy |
| 10042332 | Subclavian artery embolism |
| 10069695 | Subclavian artery occlusion |
| 10042334 | Subclavian artery thrombosis |
| 10064958 | Thromboembolectomy |
| 10043645 | Thrombotic microangiopathy |
| 10043648 | Thrombotic thrombocytopenic purpura |
| 10044390 | Transient ischaemic attack |
| 10062363 | Truncus coeliacus thrombosis |
| 10078269 | Vascular pseudoaneurysm thrombosis |
| 10048965 | Vertebral artery occlusion |
| 10057777 | Vertebral artery thrombosis |
| 10089401 | Vertebrobasilar infarction |
| 10047532 | Visual acuity reduced transiently |

# S3: The Complete List of Drugs included in the Disproportionality Analysis

| **Female hormones** |  | **NSAIDs and acetaminophen** | |
| --- | --- | --- | --- |
| Drospirenone |  | Rofecoxib | Pranoprofen |
| Ethinylestradiol |  | Acetaminophen | Tolmetin |
| Etonogestrel |  | Ibuprofen | Tolfenamic |
| Norelgestromin |  | Celecoxib | Tenoxicam |
| Desogestrel |  | Valdecoxib | Pelubiprofen |
| Megestrol |  | Ketorolac | Tiaprofenic |
| Norethisterone |  | Piroxicam | Acemetacin |
| Medroxyprogesterone |  | Naproxen | Fenbufen |
| Norgestrel |  | Diclofenac | Bendazac |
| Estradiol |  | Ketoprofen | Dexibuprofen |
| Progesterone |  | Meloxicam | Alminoprofen |
| Levonorgestrel |  | Etodolac | Esflurbiprofen |
| Estrogen |  | Loxoprofen | Phenylbutazone |
| Hydroxyprogesterone |  | Oxaprozin | Piketoprofen |
|  |  | Nabumetone | Bromfenac |
| **Male hormones** |  | Etoricoxib | Suprofen |
| Testosterone |  | Indometacin | Benoxaprofen |
| Testosterone Cipionate |  | Flurbiprofen | Furprofen |
| Testosterone Enantate |  | Mefenamic | Pirprofen |
| Testosterone Undecanoate |  | Sulindac | Fenoprofen |
| Testosterone Propionate |  | Aceclofenac |  |
| Methyltestosterone |  | Lornoxicam |  |
| Testosterone Decanoate |  | Nepafenac |  |
| Testosterone Isocaproate |  | Zaltoprofen |  |
| Testosterone Acetate |  | Dexketoprofen |  |
| Testosterone Undecylenate |  |  |  |
| Testosterone Phenylpropionate |  |  |  |
| Testosterone Hexahydrobenzoate |  |  |  |

# S4: Disproportionality Analysis focusing on a Single Drug for VTE: Representative Female and Male Hormones, and Analgesics Associated with VTE Reporting based on Crude RORs (Lower 95%CI>1.0) and χ² values

| **Female hormones** | **n_11_** | **n_12_** | **n_21_** | **n_22_** | **Crude** | **95%CI** | **χ²** |
| --- | --- | --- | --- | --- | --- | --- | --- |
|  |  |  |  |  | **ROR** |  |  |
| Drospirenone | 11,356 | 28,064 | 151,490 | 15,766,649 | 42.11 | 41.18–43.07 | 302,082.10 |
| Ethinylestradiol | 21,438 | 116,743 | 141,408 | 15,677,970 | 20.36 | 20.05–20.68 | 289,896.09 |
| Etonogestrel | 5,408 | 42,087 | 157,438 | 15,752,626 | 12.86 | 12.49–13.23 | 50,676.45 |
| Norelgestromin | 1,886 | 27,605 | 160,960 | 15,767,108 | 6.69 | 6.39–7.01 | 8,449.71 |
| Desogestrel | 969 | 10,331 | 161,877 | 15,784,382 | 9.15 | 8.56–9.77 | 6,389.51 |
| Megestrol | 694 | 12,376 | 162,152 | 15,782,337 | 5.46 | 5.06–5.89 | 2,382.67 |
| Norethisterone | 1,133 | 32,970 | 161,713 | 15,761,743 | 3.35 | 3.16–3.55 | 1,792.66 |
| Medroxyprogesterone | 1,189 | 42,534 | 161,657 | 15,752,179 | 2.72 | 2.57–2.89 | 1,252.80 |
| Estrogen | 1,273 | 63,335 | 161,573 | 15,731,378 | 1.96 | 1.85–2.07 | 579.43 |
| Norgestrel | 103 | 2,030 | 162,743 | 15,792,683 | 4.92 | 4.04–6.0 | 306.32 |
| Estradiol | 1,198 | 86,250 | 161,648 | 15,708,463 | 1.35 | 1.27–1.43 | 106.31 |
| Progesterone | 285 | 20,021 | 162,561 | 15,774,692 | 1.38 | 1.23–1.55 | 29.53 |
| **Male hormones** | **n_11_** | **n_12_** | **n_21_** | **n_22_** | **Crude** | **95%CI** | **χ²** |
|  |  |  |  |  | **ROR** |  |  |
| Testosterone | 3,704 | 42,353 | 159,142 | 15,752,360 | 8.66 | 8.37–8.96 | 22,546.61 |
| Testosterone Cipionate | 925 | 7,198 | 161,921 | 15,787,515 | 12.53 | 11.7–13.42 | 8,647.32 |
| Testosterone Enantate | 296 | 2,542 | 162,550 | 15,792,171 | 11.31 | 10.03–12.76 | 2,488.03 |
| Testosterone Undecanoate | 48 | 1,859 | 162,798 | 15,792,854 | 2.5 | 1.88–3.34 | 42.29 |
| Testosterone Acetate | 1 | 3 | 162,845 | 15,794,710 | 32.33 | 3.36–310.83 | 22.77 |
| Testosterone Propionate | 12 | 404 | 162,834 | 15,794,309 | 2.88 | 1.62–5.12 | 14.31 |
| Testosterone Undecylenate | 2 | 29 | 162,844 | 15,794,684 | 6.69 | 1.6–28.03 | 9.05 |
| **Analgesics** | **n_11_** | **n_12_** | **n_21_** | **n_22_** | **Crude** | **95%CI** | **χ²** |
|  |  |  |  |  | **ROR** |  |  |
| Rofecoxib | 2,218 | 35,108 | 160,628 | 15,759,605 | 6.2 | 5.94–6.47 | 8,972.44 |
| Acetaminophen | 15,874 | 872,951 | 146,972 | 14,921,762 | 1.85 | 1.82–1.88 | 5,460.02 |
| Ibuprofen | 5,279 | 247,822 | 157,567 | 15,546,891 | 2.1 | 2.04–2.16 | 2,889.16 |
| Celecoxib | 2,351 | 108,445 | 160,495 | 15,686,268 | 2.12 | 2.03–2.21 | 1,339.99 |
| Valdecoxib | 473 | 11,710 | 162,373 | 15,783,003 | 3.93 | 3.58–4.3 | 988.69 |
| Ketorolac | 577 | 17,266 | 162,269 | 15,777,447 | 3.25 | 2.99–3.53 | 866.29 |
| Piroxicam | 292 | 7,590 | 162,554 | 15,787,123 | 3.74 | 3.32–4.2 | 562.48 |
| Naproxen | 2,503 | 154,377 | 160,343 | 15,640,336 | 1.58 | 1.52–1.65 | 518.59 |
| Diclofenac | 2,038 | 154,117 | 160,808 | 15,640,596 | 1.29 | 1.23–1.34 | 126.47 |
| Ketoprofen | 297 | 15,343 | 162,549 | 15,779,370 | 1.88 | 1.68–2.11 | 119.61 |
| Meloxicam | 950 | 65,518 | 161,896 | 15,729,195 | 1.41 | 1.32–1.5 | 110.41 |
| Etodolac | 149 | 8,057 | 162,697 | 15,786,656 | 1.79 | 1.53–2.11 | 51.41 |
| Loxoprofen | 292 | 19,049 | 162,554 | 15,775,664 | 1.49 | 1.33–1.67 | 45.89 |
| Oxaprozin | 44 | 1,709 | 162,802 | 15,793,004 | 2.5 | 1.85 - 3.37 | 38.51 |
| Nabumetone | 149 | 8,707 | 162,697 | 15,786,006 | 1.66 | 1.41 - 1.95 | 38.44 |
| Etoricoxib | 126 | 7,168 | 162,720 | 15,787,545 | 1.71 | 1.43–2.03 | 36.11 |
| Indometacin | 237 | 16,280 | 162,609 | 15,778,433 | 1.41 | 1.24–1.61 | 28.11 |
| Flurbiprofen | 62 | 3,145 | 162,784 | 15,791,568 | 1.91 | 1.49–2.46 | 26.46 |
| Mefenamic | 71 | 4,198 | 162,775 | 15,790,515 | 1.64 | 1.3–2.07 | 17.46 |
| Sulindac | 50 | 2,946 | 162,796 | 15,791,767 | 1.65 | 1.24–2.18 | 12.47 |
| Aceclofenac | 27 | 1,475 | 162,819 | 15,793,238 | 1.78 | 1.21 - 2.6 | 8.98 |
| Lornoxicam | 16 | 796 | 162,830 | 15,793,917 | 1.95 | 1.19 - 3.2 | 7.25 |

n_11_, number of VTE events with the drug; n_12_, number of all other adverse events with the drug; n_21_, number of VTE events with other medications; n_22_, number of all other adverse events with other medications

# S5: Disproportionality Analysis focusing on a Single Drug for ATE: Representative Female and Male Hormones, and Analgesics associated with ATE Reporting based on ROR (Lower 95%CI > 1.0) and χ² values.

| **Female hormones** | **n_11_** | **n_12_** | **n_21_** | **n_22_** | **Crude** | **95%CI** | **χ²** |
| --- | --- | --- | --- | --- | --- | --- | --- |
|  |  |  |  |  | **ROR** |  |  |
| Estrogen | 2,067 | 62,541 | 281,130 | 15,611,821 | 1.84 | 1.76 - 1.92 | 755.25 |
| Drospirenone | 1,165 | 38,255 | 282,032 | 15,636,107 | 1.69 | 1.59–1.79 | 316.01 |
| Megestrol | 306 | 12,764 | 282,891 | 15,661,598 | 1.33 | 1.18–1.49 | 24.09 |
| Medroxyprogesterone | 847 | 42,876 | 282,350 | 15,631,486 | 1.09 | 1.02–1.17 | 6.64 |
| **Male hormones** | **n_11_** | **n_12_** | **n_21_** | **n_22_** | **Crude** | **95%CI** | **χ²** |
|  |  |  |  |  | **ROR** |  |  |
| Testosterone | 5,401 | 40,656 | 277,796 | 15,633,706 | 7.48 | 7.27–7.69 | 26,244.21 |
| Testosterone Cipionate | 1,434 | 6,689 | 281,763 | 15,667,673 | 11.92 | 11.26–12.62 | 11,755.26 |
| Testosterone Enantate | 282 | 2,556 | 282,915 | 15,671,806 | 6.11 | 5.4–6.91 | 1,084.74 |
| Methyltestosterone | 115 | 2,935 | 283,082 | 15,671,427 | 2.17 | 1.8–2.61 | 69.71 |
| Testosterone Propionate | 17 | 399 | 283,180 | 15,673,963 | 2.36 | 1.45–3.83 | 12.75 |
| Testosterone Acetate | 1 | 3 | 283,196 | 15,674,359 | 18.45 | 1.92 - 177.37 | 12.38 |
| Testosterone Undecanoate | 46 | 1,861 | 283,151 | 15,672,501 | 1.37 | 1.02–1.83 | 4.45 |
| **Analgesics** | **n_11_** | **n_12_** | **n_21_** | **n_22_** | **Crude** | **95%CI** | **χ²** |
|  |  |  |  |  | **ROR** |  |  |
| Rofecoxib | 15,646 | 21,680 | 267,551 | 15,652,682 | 42.22 | 41.35–43.11 | 345,852.68 |
| Valdecoxib | 3,512 | 8,671 | 279,685 | 15,665,691 | 22.69 | 21.81–23.6 | 51,185.96 |
| Celecoxib | 8,077 | 102,719 | 275,120 | 15,571,643 | 4.45 | 4.35–4.55 | 19,468.85 |
| Acetaminophen | 18,638 | 870,187 | 264,559 | 14,804,175 | 1.2 | 1.18–1.22 | 560.67 |
| Indometacin | 674 | 15,843 | 282,523 | 15,658,519 | 2.36 | 2.18–2.55 | 504.36 |
| Oxaprozin | 94 | 1,659 | 283,103 | 15,672,703 | 3.14 | 2.55 - 3.86 | 129.44 |
| Piroxicam | 265 | 7,617 | 282,932 | 15,666,745 | 1.93 | 1.7–2.18 | 113.99 |
| Etodolac | 245 | 7,961 | 282,952 | 15,666,401 | 1.7 | 1.5–1.94 | 69.06 |
| Lornoxicam | 45 | 767 | 283,152 | 15,673,595 | 3.25 | 2.4 - 4.39 | 66.11 |
| Etoricoxib | 203 | 7,091 | 282,994 | 15,667,271 | 1.58 | 1.38 - 1.82 | 42.57 |
| Naproxen | 3072 | 153,808 | 280,125 | 15,520,554 | 1.11 | 1.07–1.15 | 30.6 |
| Flurbiprofen | 87 | 3,120 | 283,110 | 15,671,242 | 1.54 | 1.25 - 1.91 | 16.19 |
| Sulindac | 80 | 2,916 | 283,117 | 15,671,446 | 1.52 | 1.22 - 1.9 | 13.79 |
| Phenylbutazone | 15 | 328 | 283,182 | 15,674,034 | 2.53 | 1.51 - 4.25 | 13.29 |
| Fenbufen | 2 | 19 | 283,195 | 15,674,343 | 5.83 | 1.36 - 25.01 | 7.23 |
| Tolmetin | 8 | 199 | 283,189 | 15,674,163 | 2.23 | 1.1 - 4.51 | 5.19 |

n_11_, number of ATE events with the drug; n_12_, number of all other adverse events with the drug; n_21_, number of ATE events with other medications; n_22_, number of all other adverse events with other medications.

# S6: Number and Proportion of Reports Involving Ethinylestradiol Among Female Hormones

| Drug A | Total | Only Drug A | Drug A and EE | Drug A and EE（％） |
| --- | --- | --- | --- | --- |
| Norelgestromin | 1,886 | 0 | 1,886 | 100 |
| Drospirenone | 11,356 | 56 | 11,300 | 99.5 |
| Norgestrel | 103 | 2 | 101 | 98.1 |
| Etonogestrel | 5,408 | 275 | 5,133 | 94.9 |
| Desogestrel | 969 | 130 | 839 | 86.6 |
| Norethisterone | 1,133 | 337 | 796 | 70.3 |
| Levonorgestrel | 1,775 | 556 | 1,219 | 68.7 |
| Medroxyprogesterone | 1,189 | 947 | 242 | 20.4 |
| Progesterone | 285 | 229 | 56 | 19.6 |
| Estradiol | 1,198 | 1,105 | 93 | 7.8 |
| Estrogen | 1,273 | 1,194 | 79 | 6.2 |
| Megestrol | 694 | 687 | 7 | 1.0 |

EE: Ethinylestradiol

# S7: A Heat map of DDI indices for VTE and ATE. Drug pairs were presented in descending order based on Ω_025_ values for VTE, including only those with Ω_025_ > 0.

|  |  |  | **VTE** | | | | |  | **ATE** | | | | |
| --- | --- | --- | --- | --- | --- | --- | --- | --- | --- | --- | --- | --- | --- |
| **DrugA** | **DrugB** |  | **n_111_** | **Ω_025_** | **Additive** | **Multi plicative** | **CRR** |  | **n_111_** | **Ω_025_** | **Additive** | **Multi plicative** | **CRR** |
| Desogestrel | Piroxicam |  | 158 | 2.54 | 0.47 | 4.33 | 6.44 |  | 0 |  | -0.03 | 0 | 0 |
| Desogestrel | Meloxicam |  | 167 | 2.39 | 0.39 | 5.4 | 5.37 |  | 1 | -5.09 | -0.01 | 0.18 | 0.15 |
| Celecoxib | Desogestrel |  | 188 | 2.05 | 0.31 | 2.77 | 4.52 |  | 5 | -3.97 | -0.06 | 0.17 | 0.14 |
| Ethinylestradiol | Ketorolac |  | 365 | 1.36 | 0.29 | 2.09 | 2.52 |  | 35 | 0.76 | 0.03 | 2.52 | 2.33 |
| Ketoprofen | Levonorgestrel |  | 9 | 1.29 | 0.09 | 5.92 | 5.78 |  | 1 | -3.26 | 0.01 | 3.65 | 0.66 |
| Ibuprofen | Norelgestromin |  | 73 | 1.26 | 0.15 | 1.7 | 3.39 |  | 5 | -1.49 | 0.01 | 2.26 | 0.94 |
| Desogestrel | Naproxen |  | 198 | 1.23 | 0.13 | 1.95 | 2.47 |  | 7 | -2.4 | -0.01 | 0.44 | 0.38 |
| Ethinylestradiol | Piroxicam |  | 190 | 1.23 | 0.27 | 1.82 | 2.43 |  | 0 |  | -0.04 | 0 | 0 |
| Norelgestromin | Paracetamol |  | 110 | 1.2 | 0.12 | 1.7 | 2.97 |  | 11 | -0.97 | 0.01 | 2.23 | 0.91 |
| Desogestrel | Diclofenac |  | 313 | 1.05 | 0.09 | 2.13 | 1.91 |  | 7 | -3.31 | -0.01 | 0.21 | 0.2 |
| Naproxen | Norelgestromin |  | 31 | 1.05 | 0.14 | 2.1 | 3.22 |  | 1 | -4.02 | 0 | 0.81 | 0.34 |
| Norethisterone | Piroxicam |  | 17 | 1.05 | 0.14 | 1.77 | 5.46 |  | 1 | -3.98 | -0.01 | 0.6 | 0.35 |
| Drospirenone | Ketorolac |  | 354 | 0.97 | 0.34 | 1.62 | 2.02 |  | 27 | 0.14 | 0.02 | 1.65 | 1.6 |
| Drospirenone | Paracetamol |  | 3,311 | 0.94 | 0.24 | 1.31 | 1.63 |  | 287 | 0.35 | 0.01 | 1.35 | 1.45 |
| Ethinylestradiol | Ibuprofen |  | 3,119 | 0.9 | 0.13 | 1.93 | 1.6 |  | 278 | 0.31 | 0.01 | 1.66 | 1.4 |
| Ibuprofen | Levonorgestrel |  | 122 | 0.9 | 0.03 | 2.31 | 2.17 |  | 22 | -1.7 | 0.01 | 2.94 | 0.52 |
| Drospirenone | Ibuprofen |  | 2,485 | 0.89 | 0.24 | 1.63 | 1.64 |  | 221 | 0.5 | 0.02 | 1.86 | 1.5 |
| Aceclofenac | Levonorgestrel |  | 3 | 0.88 | 0.41 | 26.58 | 23.84 |  | 0 |  | -0.1 | 0 | 0 |
| Acetylsalicylate | Levonorgestrel |  | 35 | 0.85 | 0.02 | 2.55 | 2.52 |  | 39 | -0.88 | 0 | 3.9 | 0.66 |
| Levonorgestrel | Paracetamol |  | 143 | 0.84 | 0.02 | 2.14 | 2.02 |  | 40 | -1.43 | 0 | 2.9 | 0.5 |
| Ethinylestradiol | Naproxen |  | 1,259 | 0.83 | 0.13 | 2.05 | 1.61 |  | 115 | 0.15 | 0.01 | 1.35 | 1.32 |
| Ethinylestradiol | Etodolac |  | 69 | 0.83 | 0.2 | 2.05 | 2 |  | 7 | -0.82 | 0.01 | 1.21 | 1.2 |
| Ethinylestradiol | Paracetamol |  | 4,162 | 0.78 | 0.11 | 1.17 | 1.49 |  | 376 | 0.01 | 0 | 1.15 | 1.1 |
| Drospirenone | Nabumetone |  | 77 | 0.78 | 0.34 | 2.5 | 2.01 |  | 6 | -1.12 | 0 | 0.81 | 1.34 |
| Drospirenone | Naproxen |  | 950 | 0.72 | 0.21 | 1.67 | 1.59 |  | 84 | 0.2 | 0.01 | 1.38 | 1.46 |
| Ethinylestradiol | Nabumetone |  | 80 | 0.72 | 0.17 | 2.3 | 1.81 |  | 8 | -1.17 | 0 | 0.89 | 0.89 |
| Drospirenone | Rofecoxib |  | 35 | 0.69 | 0.38 | 0.4 | 2.31 |  | 2 | -5.09 | -0.39 | 0.06 | 0.09 |
| Acetylsalicylate | Nomegestrol |  | 8 | 0.68 | 0.16 | 2.7 | 3.48 |  | 0 |  | -0.06 | 0 | 0 |
| Drospirenone | Etodolac |  | 57 | 0.67 | 0.31 | 1.75 | 1.94 |  | 4 | -1.39 | 0 | 0.85 | 1.41 |
| Diclofenac | Levonorgestrel |  | 17 | 0.65 | 0.02 | 2.65 | 2.64 |  | 15 | -0.01 | 0.03 | 9.58 | 1.68 |
| Celecoxib | Drospirenone |  | 176 | 0.62 | 0.22 | 0.87 | 1.68 |  | 12 | -2.03 | -0.05 | 0.28 | 0.48 |
| Drospirenone | Indometacin |  | 61 | 0.59 | 0.27 | 1.73 | 1.81 |  | 2 | -3.31 | -0.03 | 0.27 | 0.45 |
| Drospirenone | Valdecoxib |  | 21 | 0.55 | 0.38 | 0.62 | 2.27 |  | 0 |  | -0.3 | 0 | 0 |
| Celecoxib | Ethinylestradiol |  | 407 | 0.54 | 0.09 | 0.83 | 1.45 |  | 36 | -2.16 | -0.05 | 0.3 | 0.3 |
| Ethinylestradiol | Meloxicam |  | 351 | 0.51 | 0.09 | 1.54 | 1.39 |  | 18 | -1.22 | -0.01 | 0.68 | 0.68 |
| Levonorgestrel | Naproxen |  | 33 | 0.51 | 0.02 | 2.03 | 2 |  | 11 | -1.7 | 0.01 | 3.07 | 0.55 |
| Ibuprofen | Medroxyprogesterone |  | 93 | 0.5 | 0.03 | 1.18 | 2.3 |  | 55 | 0.6 | 0.02 | 2.22 | 1.92 |
| Acetylsalicylate | Drospirenone |  | 521 | 0.49 | 0.15 | 1.01 | 1.42 |  | 133 | 0.48 | 0.04 | 1.13 | 1.73 |
| Drospirenone | Meloxicam |  | 154 | 0.47 | 0.18 | 1.29 | 1.52 |  | 13 | -0.41 | 0.01 | 1.3 | 1.33 |
| Etonogestrel | Ibuprofen |  | 208 | 0.37 | 0.06 | 0.77 | 1.52 |  | 22 | -0.52 | 0.01 | 1.95 | 1.18 |
| Diclofenac | Drospirenone |  | 152 | 0.37 | 0.15 | 1.19 | 1.42 |  | 15 | -0.22 | 0.01 | 1.44 | 1.46 |
| Etonogestrel | Paracetamol |  | 271 | 0.36 | 0.05 | 0.82 | 1.46 |  | 27 | -0.83 | 0 | 1.33 | 0.81 |
| Acetylsalicylate | Ethinylestradiol |  | 649 | 0.34 | 0.06 | 0.86 | 1.22 |  | 227 | 0.18 | 0.02 | 1.25 | 1.16 |
| Ketorolac | Medroxyprogesterone |  | 13 | 0.29 | 0.06 | 1.25 | 3.26 |  | 4 | -0.8 | 0.01 | 1.64 | 1.68 |
| Ethinylestradiol | Oxaprozin |  | 17 | 0.22 | 0.15 | 1.12 | 1.75 |  | 1 | -4.06 | -0.04 | 0.33 | 0.34 |
| Medroxyprogesterone | Paracetamol |  | 169 | 0.21 | 0.01 | 0.96 | 1.64 |  | 164 | 0.83 | 0.02 | 2.14 | 2.05 |
| Drospirenone | Flurbiprofen |  | 8 | 0.2 | 0.43 | 1.42 | 2.35 |  | 1 | -2.13 | 0.05 | 2.03 | 3.07 |
| Acetylsalicylate | Etonogestrel |  | 65 | 0.19 | 0.05 | 0.99 | 1.46 |  | 26 | -0.31 | 0.02 | 1.78 | 1.05 |
| Norethisterone | Paracetamol |  | 209 | 0.16 | 0.01 | 0.88 | 1.48 |  | 42 | -1.51 | 0 | 0.79 | 0.47 |
| Ethinylestradiol | Valdecoxib |  | 29 | 0.16 | 0.1 | 0.44 | 1.61 |  | 4 | -4.16 | -0.25 | 0.13 | 0.13 |
| Acetylsalicylate | Norelgestromin |  | 18 | 0.16 | 0.05 | 1.29 | 1.91 |  | 9 | -0.85 | 0.01 | 2.35 | 0.95 |
| Ibuprofen | Norethisterone |  | 118 | 0.14 | 0.01 | 0.85 | 1.66 |  | 27 | -1.02 | 0 | 1.37 | 0.79 |
| Desogestrel | Ibuprofen |  | 306 | 0.13 | 0.02 | 0.68 | 1.24 |  | 1 | -8.06 | -0.02 | 0.02 | 0.02 |
| Medroxyprogesterone | Naproxen |  | 44 | 0.1 | 0.01 | 1.12 | 1.71 |  | 33 | 0.25 | 0.01 | 1.68 | 1.79 |
| Ethinylestradiol | Indometacin |  | 67 | 0.06 | 0.05 | 1.14 | 1.17 |  | 5 | -2.6 | -0.03 | 0.37 | 0.38 |
| Celecoxib | Norelgestromin |  | 8 | 0.05 | 0.09 | 1.21 | 2.53 |  | 2 | -2.7 | -0.02 | 1.31 | 0.55 |
| Megestrol | Rofecoxib |  | 10 | 0.02 | 0.09 | 0.62 | 3.2 |  | 12 | -1.66 | -0.19 | 0.41 | 0.52 |
